# Letter from Dr. Thomas W. Evans of Paris

**Published:** 1869-05

**Authors:** Thomas W. Evans


### CORRESPONDENCE.

### ARTICLE V.

15 RUE DE LA PAIX,
PARIS, *Mch.* 16, 1869.

*To the Editor of the American Journal of Dental Science.*

SIR:—

My attention has been directed to an article entitled "*A Novel Invention,*" published in the Editorial Department of the February number of "*The American Journal of Dental Science.*"

The occasion for some rather severe criticism is to be found in the following passage reproduced from "L'Art Dentaire."

"Mr. Evans proposes a new method of using nitrous oxide as an anæsthetic. It is administered in liquid state, and as it evolves itself in gas in the interior of the stomach it will produce the desired insensibility."

Such is the *reported* substance of a communication presented by me to the *Academie des Sciences!*

I am not so fond of error even of my own creating, as to be unwilling to receive a merited correction. But in this case, I must confess I sincerely regretted to see the "*Journal*" betrayed, by following the lead of an obscure publication into a Quixotic tilt against—nothing.

The fact is, the paragraph you translate so far as it purports to give a proposition of mine is entirely without foundation. No such proposition was ever made by me—no such idea was ever entertained. In the paper upon "Liquid Protoxide of Azote" which I submitted to the *Academie des Sciences* last August, after having described the process employed for liquifying the gas, I said— "The gas thus liquefied was drawn off through an escape pipe into a caoutchouc bag where it rapidly resumed its original volume and from which it was administered to the patient in the usual way." And again—"Besides, using the fluid protoxide of azote as a general anæsthetic by inhalation—availing myself of its great refrigerating power I have used it in several instances to produce local anæsthesia. This can be accomplished upon any surface of the body by simply directing upon it for a moment the jet of vaporous gas as it escapes from the nozzle of the bottle."

The paper closes as follows:—"In a word, I have shown, 1st, that liquid protoxide of azote may be used as a general anæsthetic by inhalation, with the advantage over the gas in its ordinary form of greater purity, compactness and portability. 2d, that the spray of liquid of protoxide of azote is a most powerful and efficient local anæsthetic, with the advantage over other local anæethstics of greater certainty in its effects as also, that it may be applied without a special apparatus."

I presume it is hardly necessary that I should say more upon this subject--and I only regret that it has seemed necessary that I should say so much, to prove to the "*Journal*" and its readers the falsity of a statement, the improbability of which—I should have supposed, was sufficiently evident upon its face.

Yours very truly,

THOMAS W. EVANS, M.D.

