# Correction. Dr. Thos. W. Evans

**Published:** 1869-05

**Authors:** 


*Correction—Dr. Thomas W. Evans.*—Under "Correspondence" our readers will find a letter from Dr. Thomas W. Evans of Paris, to which we invite their attention.

We are much gratified in being able to do justice to Dr. Evans, as we feel that the editorial in the Feb. number of the *Journal*, of which he justly complains, has placed him in a false position.

The paragraph which led to the preparation of the editorial in question, was translated from the French periodical known as the *L'Art Dentaire*, and our readers will observe that Dr. Evans denies that any such proposition, as is therein ascribed to him, was ever made by him.

We at all times endeavor to do justice, and sincerely regret that the *Journal* has been betrayed into an act of injustice towards one to whom the dental profession owes so much for his untiring energy in its behalf.

